# Advertisement by medical facilities as an opportunity route of *APOE* genetic testing in Japan: a website analysis

**DOI:** 10.1007/s12687-024-00697-9

**Published:** 2024-01-16

**Authors:** Kenichiro Sato, Yoshiki Niimi, Ryoko Ihara, Atsushi Iwata, Takeshi Ikeuchi, Takeshi Iwatsubo

**Affiliations:** 1 Department of Neuropathology, Graduate School of Medicine, The University of Tokyo, Hongo 7-3-1, Bunkyo-Ku, Tokyo, 113-8655 Japan; 2 Unit for Early and Exploratory Clinical Development, The University of Tokyo Hospital, Hongo 7-3-1, Bunkyo-Ku, Tokyo, 113-8655 Japan; 3 Department of Neurology, Tokyo Metropolitan Institute for Geriatrics and Gerontology, Sakaecho 35-2, Itabashi-Ku, Tokyo, 173-0015 Japan; 4 Department of Molecular Genetics, Brain Research Institute, Niigata University, Asahimachidori 1-757, Chuo-Ku, Niigata, 951-8585 Japan

**Keywords:** *APOE*, Genetic testing, Advertisements, Website analysis, Alzheimer’s disease

## Abstract

The *APOE*-ε4 allele(s) is a strong risk factor for Alzheimer’s disease (AD). A significant point of access for this allele testing is through services provided by medical facilities in Japan, which advertise out-of-insurance APOE testing on their websites. There is a concern that website advertisements for *APOE* testing may influence the ability for individuals to adequately self-determine whether to undergo *APOE* testing. We conducted a cross-sectional survey on medical facility websites in Japan advertising *APOE* genetic testing. We predefined desirable features for advertisement descriptions based on legal regulations and guidelines published by relevant professional societies and evaluated each website according to these features. We identified 220 medical facilities that had posted advertisements on their websites for the provision of *APOE* genetic testing, of which 85% were small clinics. Contact information, details, and costs of testing were described in most of the websites. Meanwhile, features such as “explaining *APOE* as a risk gene,” “notes on interpreting *APOE* results,” or “explaining examination methods” (e.g., blood sampling) were described to a variable degree depending on individual facilities. “Notes on genetic testing” or “referring to genetic counseling” were hardly referred to, and specialists with appropriate expertise were considered to participate in clinical practice in approximately one-third of these facilities providing *APOE* testing services. These website evaluation results showed moderate to substantial reliability between independent raters. These results suggest that self-determination of pursuing out-of-insurance *APOE* testing at some medical facilities in Japan may possibly be influenced in an inappropriate manner, at least in its entry route of taking the test.

**Supplementary Information:**

The online version contains supplementary material available at 10.1007/s12687-024-00697-9.

## Background

The presence of the *APOE*-ε4 allele(s) is a strong risk factor related to the development of Alzheimer’s disease (AD) (e.g., odds ratio (OR) 5.9 in ε4/ε3 and OR 33.1 in ε4/ε4 in Japanese population) (Liu et al. 2013), especially of late-onset AD. Therefore, it is essential in the clinical research of AD. Additionally, the use of anti-amyloid antibodies as disease-modifying therapies (DMTs) for AD has been linked to an increased risk of developing amyloid-related imaging abnormalities, including edema/effusion (ARIA-E) and hemorrhage (ARIA-H), which are specific side effects of these treatments (Sperling et al. 2011). Drugs such as aducanumab and lecanemab, which have received FDA approval, illustrate this association (Dhillon 2021). This means the *APOE* genotype is also important information in clinical practice when anti-amyloid antibodies become widely available (Cummings et al. 2021), for example as a companion diagnostic. Appropriate use recommendations of lecanemab published in 2023 actually recommend *APOE* genotyping of all treatment candidates to assess the risk of ARIA before starting treatment with lecanemab (Cummings et al. 2023).

However, the contribution of *APOE* genotype to the development of AD is at best medium-to-high, unlike the definitive contribution of genetic mutations such as *PSEN-1*, *PSEN-2*, or *APP* in familial AD (Nicolas et al. 2016). And the clinical impact of DMT in the suppression and prevention of AD remains under investigation. Based on these characteristics of the *APOE* genotype, as of early 2023, *APOE* genetic testing has not been covered by health insurance in many countries (Arias et al. 2021), including Japan. Clinical practice guidelines for dementia in Japan (Japanese Society of Neurology n.d.) published in 2017 have not recommended routine testing of the *APOE* genotype in daily clinical practice for dementia care in medical facilities. It is only since the recent approval of lecanemab in 2023 that *APOE* testing is to be considered in clinical practice.

Meanwhile, out-of-insurance testing or direct-to-consumer (DTC) testing of *the APOE* genotype has been available (Pavarini et al. 2021), especially for those who are cognitively normal but are worried about their risk of dementia to prepare in advance before developing dementia. While the current clinical guidelines do not always positively support *APOE* testing services, they may have potential benefits leading to positive health-related behaviors (Marshe et al. 2019), and they should not be restricted from the aspect of individual patients’ right to know their own genetic risk of having AD in the future. Furthermore, disclosing the *APOE* genotype after careful and sufficient information distribution does not always result in adverse consequences (e.g., increased anxiety or depression levels) in clinical research settings (Green et al. 2009; Bemelmans et al. 2016; Alber et al. 2022). However, conversely, it may have the potential risk of causing harm to individuals and their relatives (e.g., test-related distress (Green et al. 2009; Bemelmans et al. 2016; De et al. 2021)) if examined and disclosed inappropriately, e.g., without conveying sufficient information. Thus, *APOE* testing should only be conducted after sufficient explanation within clinical practice settings.

As of August 2023, *APOE* genetic testing has not always been familiar in Japan, a country facing the significant challenge of having the highest aging rate (Muramatsu and Akiyama 2011). This challenge is compounded by the increasing prevalence of dementia; for instance, it is projected that more than 20% of Japanese individuals aged 65 or older will have dementia by 2030 (Nakahori et al. 2021), highlighting the urgency of these social issues. The prevalence of *APOE* genotype among non-demented elderly Japanese individuals is reported to be 16.5% for ε3/ε4 and 1.1% for ε4/ε4 (Kobayashi et al. 2011), rates that are similar to those observed in other East Asian countries (Corbo et al. 1999). We assume that more medical facilities in Japan than before are providing testing as one of their out-of-insurance services, although detailed statistics are not available. Therefore, the websites of such medical facilities might be one of the primary sources of opportunities for *APOE* testing. The description of medical services, including examinations to attract patients to receive the services, on the website of individual medical facilities in Japan is legally regarded as an advertisement and is therefore strictly regulated by Japanese law (cf. Medical Care Act: https://www.japaneselawtranslation.go.jp/en/laws/view/4006). By this law, medical facilities cannot make advertisements for affairs other than the prespecified items (positive list) (cf., Medical Care Act, Article 6–5-3), such as examinations being covered by health insurance. In terms of medical services not corresponding to the positive list, this regulation is to be released if the specified descriptions are adequately equipped on the website pages (cf., Enforcement Regulations on the Medical Care Act, Article 1–9-2-iii, iv), as follows: (a) contact information of medical facilities (e.g., phone or e-mail address), (b) “information on particulars about the details and costs of treatment, etc., normally required for medical not covered by health insurance,” and (c) “information on particulars about major risks and adverse reactions, etc., by medical care not covered by health insurance.” Above (b) and (c) are applicable only for out-of-insurance services. Based on the above legal requirements, posting an explanation or description of *APOE* testing on the websites of medical facilities might be needed.

Although there should be no clear criteria as to the required degree of content of *APOE* genotype testing information to be provided, the website advertisement is believed to be one of the key opportunity routes to testing, which means that adequate self-determination of whether to take *APOE* testing may be lacking if the information is provided inappropriately. Thus, here, we investigated the extent to which information is provided on the website of each medical facility advertising *APOE* testing in Japan by conducting a website analysis of medical facilities (Kashihara et al. 2016; Taylor-Phillips et al. 2020).

## Methods

### Study purposes and prerequisites

In Japan, *APOE* testing can be performed through several avenues, including out-of-insurance service at medical facilities, clinical research, clinical trials of AD treatment, or DTC services. However, these options are not always commonly utilized by the Japanese people. Detailed data on the proportion of individuals who have undergone *APOE* testing, and through which specific avenues, is not available. In this study, we conducted website review surveys of *APOE* genetic testing advertisement content. Our institutional review board approved this study (ID: 11628-(3)). Informed consent was not required because individual patient data were not used here. All authors are academic researchers and clinical neurologists without specific conflicts of interest related to *APOE* genetic testing services and are not personnel of the government office in charge of advertisement regulation. Therefore, we are not legally authorized to judge whether individual website advertisements are conducted following the current law and guidelines for medical advertisements (Ministry of Health, Labour, and Welfare n.d.); this is a prerequisite for this study. Thus, we aimed to analyze and discuss the overall tendency of advertisements for *APOE* testing by medical facilities in Japan rather than to review and discuss individual websites in an identifiable manner. In addition, we will not publicize the list of medical facilities we analyzed in an identifiable manner.

### Website searching

We manually searched the websites of individual medical facilities in Japan by popular Internet search engines in Japan (e.g., Google, Yahoo! Japan, and Bing) in April 2022, by the search terms, which are a combination of words “APOE,” “genetic testing,” and prefecture name in the corresponding Japanese words. Only websites written in Japanese were included, and a few websites for which medical facilities or their clinicians were unidentifiable were excluded in advance because descriptions on such websites are not defined as medical advertisements by the law. Identified websites were then manually examined to code whether they introduced *APOE* genetic testing as one of the medical services provided in the facility. We excluded facility websites that only referred to the unspecific names of the testing (e.g., “genetic testing related to dementia”), those explicitly referring to APOE protein testing only, and those clarifying not providing *APOE* testing service. We complimented the manual search by referring to the list of “affiliated medical facilities” publicized on the websites of some commercial companies providing *APOE* genetic testing services for research use. One of the authors (K. S.) primarily conducted the website search procedure, which another author (Y. N.) complimented: these authors (K. S. and Y. N.) cooperatively determined the decision on an individual website to include in the analysis.

### Extracting website characterizations

Before initiating a manual search, we specified the preferred characteristics of website descriptions concerning *APOE* testing and their definitions, as outlined in Table 1, following a discussion between K. S. and Y. N. These website features were categorized into two levels (Tier 1 and Tier 2) based on the necessity for inclusion in the website description: the Tier 1 features are those considered to be more directly associated with the legal requirements. Tier 2 features are derived from the guidelines for medical advertisement (Ministry of Health, Labour, and Welfare n.d.), dementia treatment (Japanese Society of Neurology n.d.), genetic testing in medical practice (The Japanese Association of Medical Sciences n.d.), ethical guidelines for medical research (Ethical guidelines for medical and biological research involving human subjects n.d.), or items that we consider might be important for clinicians to inform patients carefully before conducting a test.

**Table 1:** Website features and their definitions

Tier (no.)	Feature	Definition	Laws/guidelines we referred to
1–1	Contact information	Telephone and e-mail addresses are available on the website	Medical Care Act, (Ministry of Health, Labour, and Welfare n.d.)
1–2	Details *or* costs of testing	Either Tier 2–1 *or* 2–2 is satisfied	Medical Care Act, (Ministry of Health, Labour, and Welfare n.d.)
2–1	Explaining the *APOE* as a risk gene	Explaining that *APOE* testing is related to the development of AD/dementia or its risk	(Ministry of Health, Labour, and Welfare n.d.)
2–2	Cost clarified	The cost of *APOE* testing by itself is clarified	(Ministry of Health, Labour, and Welfare n.d.)
1–3	Major risks *or* adverse reactions	Either Tier 2–3 *or* 2–4 is satisfied	Medical Care Act, (Ministry of Health, Labour, and Welfare n.d.)
2–3	Notes on genetic testing	Referring to general notes on genetic testing (e.g., potential negative impact because of disclosure)	(Ministry of Health, Labour, and Welfare n.d.)
2–4	Examination method	The examination method (e.g., blood sampling) is explained	(Ministry of Health, Labour, and Welfare n.d.)
2–5	Notes on interpreting results	Referring to the notification in interpreting *APOE* testing results (e.g., “ε4-positive does not always predict future AD development,” or “ε4-negative does not always predict you are free from AD development”)	(The Japanese Association of Medical Sciences n.d.; Ethical guidelines for medical and biological research involving human subjects n.d.)
2–6	Appropriate specialists	Clinical specialists with appropriate expertise to conduct *APOE* testing are enrolled in the practice at the facility	(Japanese Society of Neurology n.d.; The Japanese Association of Medical Sciences n.d.; Ethical guidelines for medical and biological research involving human subjects n.d.)
2–7	No external links	The description of *APOE* testing on the website does not include a link to an external website for further explanations	(Ministry of Health, Labour, and Welfare n.d.)
2–8	Referring to genetic counseling	Referring to genetic counseling as per need before or after *APOE* testing	(Japanese Society of Neurology n.d.; The Japanese Association of Medical Sciences n.d.; Ethical guidelines for medical and biological research involving human subjects n.d.)
2–9	Not covered by health insurance	Clarifying the *APOE* testing is out-of-insurance or one of the medical checkup menu	(Ministry of Health, Labour, and Welfare n.d.)

Abbreviations: *AD* Alzheimer’s disease

Tier 1 features (e.g., 1–2 and 1–3) were defined as eligible when their corresponding Tier 2 features are fulfilled. For instance, Tier 1–2 is considered satisfied when *either* Tier 2–1 or 2–2 is met (i.e., logical sum), but it is not exclusively when *both* Tier 2–1 and 2–2 are met (i.e., logical conjunction) as outlined in the English translation of the Article (specifically, the Enforcement Regulations on the Medical Care Act, Article 1–9-2, referred to in subsections (b) and (c) of the “Background” section). This approach is adopted because the original Japanese text of the Article does not explicitly state that all subitems must be satisfied. Moreover, by adopting a less stringent definition, we can reduce the risk of overestimating the frequency of facilities failing to meet the criteria. Consequently, there may be some discrepancies between the defined requirements of this study and those enforced in practice. We acknowledge such discrepancies as unavoidable, given our role is not to adjudicate the compliance of individual website advertisements with the legal standards in their entirety, particularly as we lack the legal authority or capacity to do so.

### Definition in evaluating website features

Evaluation of individual websites whether they met the Tier 1 and Tier 2 features was primarily conducted by one author (K. S.). To assess the validity of the coding process, 10% of the websites (Lombard et al. 2005) were randomly selected for independent review by the second author (Y.N.). Kappa coefficients were calculated to measure inter-rater reliability. The sample size for the websites was determined based on earlier literature (Cantor 1996), using R package {*irr*} and assuming a positive rating probability of 0.5, a true kappa value of 0.7, a kappa value under the null hypothesis of 0.2, an alpha of 0.05, and a power of 0.8. The kappa coefficient and its 95% confidence interval (CI) were computed using R package {*psych*}. Kappa values are interpreted as follows: ≤ 0.2 indicates slight reliability, 0.21–0.4 fair, 0.41–0.6 moderate, 0.61–0.8 substantial, and > 0.8 almost perfect reliability, respectively (Chmura Kraemer et al. 2002). Furthermore, kappa coefficients for features that displayed zero variance in the rating results from one rater were not adequately calculable; therefore, we resorted to using agreement percentages (%) between raters for such features. This represents the proportion of facilities that both raters assessed consistently.

The feature “explaining the *APOE* as a risk gene” (Tier 2–1) was broadly defined to include phrases such as “risk gene of AD” or “genetic test related to dementia” as eligible descriptions. The cost feature (Tier 2–2) required the *APOE* testing price to be clearly stated in Japanese Yen (JP¥) and aggregated with other testing costs.

The “notes on genetic testing” feature (Tier 2–3) was considered satisfied if the advertisement addressed the potential negative impacts on patients or their relatives upon disclosure of *APOE* test results. The “examination method” feature (Tier 2–4) is determined based on whether the sampling method (e.g., blood, hair, or saliva) is clarified. The feature “notes on interpreting results” (Tier 2–5) was deemed satisfied when the advertisement description included notification sentences such as “having *APOE*-ε4 allele[s] does not surely predict future development of AD” or “not having *APOE*-ε4 allele[s] does not ensure the individual is free from dementia.”

Regarding features about specialists (Tier 2–6), on most outpatient clinic websites, clinicians’ names, careers, and specialties are also disclosed within the bounds permitted by law (cf. Medical Care Act, Article 6–5-3-ix). We regarded the *APOE* testing as adequately explained and conducted by specialists with relevant expertise if the clinicians listed on the outpatient clinic’s website were certified in any of the following fields of expertise: clinical neurology (as certified by the Japanese Society of Neurology: https://www.neurology-jp.org/en), dementia treatment (as certified by Japan Society for Dementia Research: https://square.umin.ac.jp/dementia/eng.html and Japanese Psychogeriatric Society: http://www.rounen.org), clinical psychiatry (as certified by The Japanese Society of Psychiatry and Neurology: https://www.jspn.or.jp/modules/english/index.php?content_id=1), geriatrics medicine (as certified by The Japan Geriatrics Society: https://www.jpn-geriat-soc.or.jp/en/), or clinical neurosurgery (as certified by The Japan Neurosurgical Society: https://jns-official.jp/english/about). Meanwhile, medical geneticists certified by Japanese Board of Medical Genetics and Genomics, Clinical Genetics (http://www.jbmg.jp) were not considered a relevant field of expertise. This exclusion is due to the limited number of clinics in Japan that primarily practice genetics. Additionally, certified geneticists are deemed less likely to conduct out-of-insurance *APOE* testing, particularly in the circumstances where DMT drug have not been approved and *APOE* testing is not advised by the current guidelines (Japanese Society of Neurology n.d.).

Regarding advertisement without external links (Tier 2–7), this feature was defined because the guideline on a medical advertisement recommends that individual medical facilities avoid linking to external websites, instead providing explanations directly on their own websites (Ministry of Health, Labour, and Welfare n.d.). Genetic counseling is as follows (Tier 2–8): The Japanese guidelines for genetic testing (The Japanese Association of Medical Sciences n.d.; Ethical guidelines for medical and biological research involving human subjects n.d.) state that genetic counseling should include (1) interpretation of family and medical history to assess the likelihood of disease occurrence and recurrence; (2) education on genetic phenomena, testing, management, prevention, resources, and research; (3) promotion of informed and autonomous decision-making; and (4) assistance with understanding and adjusting to the medical, psychological, and familial implications of genetic factors in diseases. While these guidelines advise clinicians or researchers to consider the necessity of genetic counseling, it is not always obligatory to perform genetic counseling for any aspects of the aforementioned processes according to the current guidelines. Nonetheless, we tailored this criteria with reference to other clinical research on AD that provides counseling for *APOE* testing (Langlois et al. 2019).

### Statistical analyses

All analyses were conducted using R 4.1.2. We first examined the proportion of websites that met each feature in Table 1. We also examined any differences in the degree of satisfaction between the types of medical facilities, namely, large hospitals (defined by the Medical Care Act, Article 1–5-1, as facilities for the hospitalization of not less than 20 patients) and small clinics (described in the Medical Care Act, Article 1–5-2, as facilities with no in-patient capacity or for the hospitalization of no more than 19 patients). We then analyzed whether these websites can be classified into several clusters with similar characteristics in their description in the *APOE* testing advertisement using latent class analysis (LCA) (Sinha et al. 2021). Adequate number of classes were determined based on the Bayesian information criterion (BIC) value. LCA was conducted using the R package {*poLCA*} (Linzer and Lewis 2011).

## Results

### Overall summary

In total, we identified 220 medical facilities that posted advertisements for the provision of *APOE* genetic testing on their websites on April 2022. Of these, 85% were small clinics, and the remaining 15% were outpatient departments of large hospitals. The top 5 locality prefectures for these facilities were Tokyo, Osaka, Kanagawa, Saitama, and Aichi, in decreasing frequency: these are the prefectures with the largest populations in Japan. The median price of *APOE* testing including VAT (when clarified) was JP¥ 17,600 [IQR, 16,500–20,000] (= US$ 123 [IQR, 115–131]: US$ 1 = JP¥ 143).

### Coding results

The feature characteristics of all websites are summarized in Table 2. Kappa coefficients between the raters are also shown. Contact information (Tier 1–1) was provided by all facilities (100%), and “details or costs of testing” (Tier 1–2) were available in most of the facilities (94.5%), exhibiting moderate inter-rater reliability with a relatively wide 95% CI (kappa 0.412, 95% CI 0.014 ~ 0.808). “Major risks and adverse reactions” (Tier 1–3) was met only in half of the facilities (50.0%) with substantial reliability (kappa 0.737, 95% CI 0.419 ~ 0.914).

**Table 2:** Result summary of website features

Tier (no.)	Feature	Satisfying websites, frequency (%)	Kappa (95% CI) (or agreement (%))
1–1	Contact information	*n* = 220 (100%)	Agreement^†^, 100%
1–2	Details *or* costs of testing	*n* = 208 (94.5%)	0.412 (0.014 ~ 0.914)
2–1	Explaining the *APOE* as a risk gene	*n* = 175 (79.5%)	0.400 (0.094 ~ 0.706)
2–2	Cost clarified	*n* = 154 (70%)	1.000 (1.000 ~ 1.000)
1–3	Major risks *or* adverse reactions	*n* = 110 (50%)	0.737 (0.419 ~ 0.914)
2–3	Notes on genetic testing	*n* = 1 (0.5%)	Agreement^†^, 100%
2–4	Examination method	*n* = 110 (50%)	0.737 (0.419 ~ 0.914)
2–5	Notes on interpreting results	*n* = 44 (20%)	1.000 (1.000 ~ 1.000)
2–6	Appropriate specialists	*n* = 78 (35.5%)	0.783 (0.517 ~ 0.929)
2–7	Not external links	*n* = 199 (90.5%)	Agreement^†^, 91%
2–8	Referring to genetic counseling	*n* = 2 (0.9%)	Agreement^†^, 100%
2–9	Not covered by health insurance	*n* = 162 (73.6%)	0.615 (0.170 ~ 0.875)

Please note that kappa coefficients of features with zero variance by one side of rater cannot be calculated adequately. For such features (as marked with a dagger (^†^)), we used agreement percentages (%) between raters instead of kappa coefficient

Abbreviations: *N/A* not available, *CI* confidence interval

Among the Tier 2 features, the examination method (Tier 2–4) was referred to in 50% of the facilities whether it is blood, hair, or mucosal sampling. “Notes on interpreting *APOE* testing results” (Tier 2–5) were referred to only in 20% of the facilities, with excellent reliability. “Notes on genetic testing” (Tier 2–3) or genetic counseling (Tier 2–8) were rarely referred to (0.5% and 0.9%, respectively), and their agreement percentage was 100%. In addition, appropriate specialists were enrolled (Tier 2–6) only in approximately one-third of all examined facilities (35.5%), with a substantial reliability (kappa 0.783, 95% CI 0.517 ~ 0.929). Referring to not being covered by health insurance (Tier 2–9) was observed in many facilities (73.6%), with a substantial reliability (kappa 0.615, 95% CI 0.170 ~ 0.875).

We then examined the differences in the proportion of features between smaller clinic and larger hospitals as shown in Supplemental Table 1. Appropriate specialists (Tier 2–6) and the testing not covered by health insurance (Tier 2–9) were more likely to be observed in larger hospitals than in smaller clinics (*p* < 0.001 and *p* = 0.003, respectively).

### Website classification by latent class analysis

**Fig. 1:**
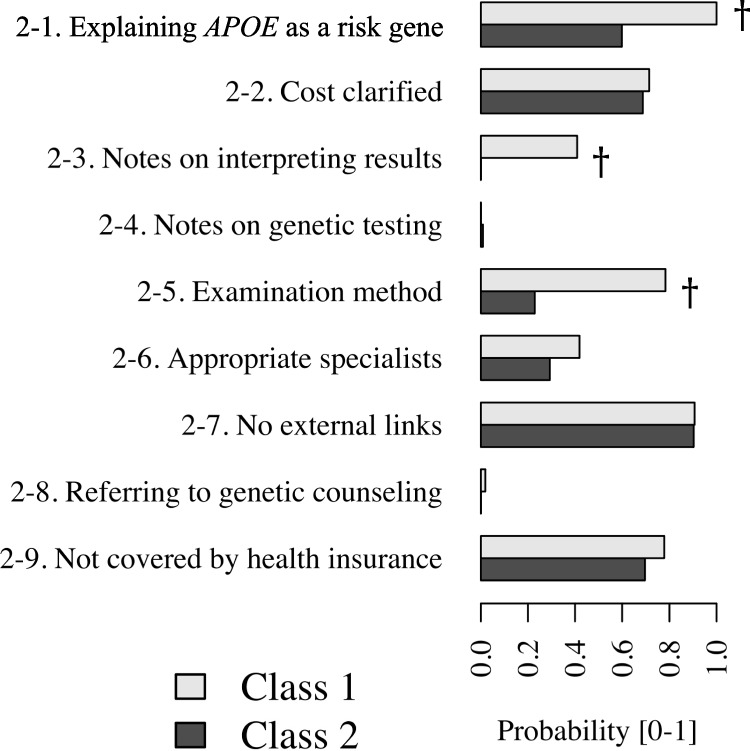
Latent class analysis results. The number of classes was determined to be two because BIC has a minimum value. The appearance probabilities of each feature in classes 1 and 2 are represented. In some features highlighted with daggers (†), there are discrepancies in the probabilities between classes 1 and 2. Abbreviations: BIC, Bayesian information criterion

Next, we conducted LCA. The optimal number of latent classes was determined to be two, based on the lowest BIC value. Figure 1 illustrates the probability of observing Tier 2 features within classes 1 and 2. While many features exhibited comparable probabilities of appearance in both classes, certain features—marked by daggers—demonstrated notable differences. Class 1 had higher probabilities for features Tier 2–1, 2–3, and 2–5, indicating that these features are more prevalent on websites classified into class 1 compared to those in class 2.

## Discussion

We analyzed the websites of 220 medical facilities advertising *APOE* genetic testing in Japan. As a result, most websites met the following regulatory requirements for posting *APOE* testing advertisements: contact information, details, testing costs, and a statement indicating that the tests are not covered by health insurance. The description of “examination methods” (e.g., blood sampling) or “notes on interpreting *APOE* results” varied significantly among facilities. References to “notes on genetic testing” and “genetic counseling” were infrequently mentioned, and “specialists with appropriate expertise” were reported to be involved in clinical practice in approximately one-third of these facilities providing *APOE* testing services. These results suggest that self-determination and its quality of *APOE* testing at some of these medical facilities in Japan could potentially be compromised, at least regarding patients’ initial decisions on whether to undergo testing.

Although all facilities met the contact information criterion (Tier 1–1) and the majority also provided details or costs of *APOE* testing (Tier 1–2), which are legal requirements for lifting restrictions on medical advertisements, with compliance rates of 100% and 94.5%, respectively, we should not overemphasize the findings of Tier 1–2 or Tier 2–1. This caution is due to our study’s aim, which was not to rigorously assess each individual website advertisement’s compliance with legal requirements. Furthermore, the observed reliability was only moderate to fair, and the lower bounds approached zero, potentially resulting from our broad definition of eligibility for Tier 2–1 in the manual search.

Tier 1–3 criteria were met in only half of the facilities with substantial reliability. This may be attributable to two reasons: (i) the possible risk of harm to patients (e.g., increased anxiety or depression levels (Green et al. 2009; Bemelmans et al. 2016; Alber et al. 2022)) following the disclosure of results may not be sufficiently acknowledged, and (ii) the invasiveness of specimen sampling for *APOE* testing (e.g., sampling of blood, hair, or buccal mucosa) is limited. According to the English translation of the Medical Care Act by the Ministry of Justice, legal requirements for lifting advertising restrictions include providing “information on particulars pertaining to *major* risks and adverse reactions, etc., by medical care not covered by health insurance.” Although the harmful risk level following *APOE* result disclosure may not be “major,” if such risks are under-recognized or overlooked by clinicians, they may overlook the necessity that they should describe its risks and adverse effects on their websites.

Approximately one-third of the facilities advertising *APOE* testing reportedly involved specialists with the appropriate expertise (Tier 2–6). This figure might be an underestimation since the determination of specialist involvement was based solely on the presence of clinician names on the website, without considering their actual employment status. Furthermore, such a limited proportion of specialists involved raises concerns about the adequacy of explanations regarding the testing’s significance and whether notes on genetic testing (Tier 2–3) are provided to patients before and at the time of result disclosure. Moreover, our findings suggest that dementia experts may be more hesitant to conduct *APOE* testing compared to their counterparts, inferred from the lower proportion of specialists versus non-specialists and the general clinician demographic in Japan. This reluctance may be informed by clinical practice guidelines for dementia that do not advocate routine *APOE* testing (Japanese Society of Neurology n.d.) and by another set of guidelines that recommend *APOE* testing after thorough consideration of its validity and utility (The Japanese Association of Medical Sciences n.d.; Ethical guidelines for medical and biological research involving human subjects n.d.). Similarly, a hesitance among genetic specialists to engage in *APOE* testing is also suspected; geneticists were rarely seen during our website review, although we have not noted them statistically. This indicates that a significant amount of out-of-insurance *APOE* testing at Japanese medical facilities may occur without the involvement of genetic specialists.

Genetic counseling was infrequently mentioned (Tier 2–8, 0.9%). It is indeed not required to be posted by the guideline of medical advertisements and is also not always required to be provided in the actual practice by the current guidelines on genetic testing (The Japanese Association of Medical Sciences n.d.; Ethical guidelines for medical and biological research involving human subjects n.d.) where clinicians or researchers are only to consider the necessity of genetic counseling for testing of susceptibility genes. However, because the proportion of specialists enrolled was relatively low, the necessity may not always be considered appropriately, which is another matter of concern. Furthermore, the shortage of medical facilities offering genetic counseling in Japan, for a range of conditions beyond *APOE*, could be a contributing factor to this deficiency.

The advertisement on *APOE* testing is occasionally accompanied by descriptions of benefits of testing, such as “*APOE* genetic testing is useful in understanding the future risk of dementia to prepare for its prevention.” However, we did not analyze such appealing expressions here. The clinical utility of drug intervention with DMT to prevent AD or dementia, or the clinical utility of multifactorial interventions, including management of vascular risk factors (e.g., hypertension, diabetes, and dyslipidemia), nutritional guidance, and exercise training (Ngandu et al. 2015; Solomon et al. 2018), has not been established so far. We are concerned that advertisements describing benefits of *APOE* testing may be overstated, depending on how they are described.

Our study had several potential shortcomings. First, we do not know how *APOE* testing is explained and conducted in actual clinical practice in each facility. The poor explanation in the website advertisement does not always mean that the in-person information provided to patients is poor. In addition, we examined facilities that made advertisements on their websites, so we could not examine those providing the testing service without doing website advertisement. In addition, this study focuses on the appropriateness of the advertisement, which has limitations in its scope in discussing how to support well-informed self-determination on whether to take the test.

Second, although we had to rely on publicized certifications to distinguish individual clinicians’ expertise, clinicians with a certification in the specific expertise areas related to dementia treatment may not always be familiar with *APOE* testing. In addition, even in the case of clinicians without specific certification testing, the actual conditions may differ between general internal medicine clinics and out-of-insurance beauty clinics, which we could not examine here.

Third, the inter-rater reliability for some features was not consistently sufficient, especially concerning “explaining as a risk gene” (i.e., Tier 1–2, 2–1), which may be because of its broadly defined yet somehow unspecified criteria. We may need to refine definitions, or to introduce another feature with a narrower scope. Additionally, adjusting the number of sampled websites to evaluate the kappa coefficient may be warranted, given that the assumptions made in the sample size calculation may not be robust. Incorporating adjustments for demographic factors, such as location or years of experience, may also be beneficial during sampling.

Lastly, we were unable to assess the quality of *APOE* testing provided by individual medical facilities, as such information is not ascertainable through website descriptions. The testing’s accuracy, which plays a crucial role in the interpretation of *APOE* results, may vary among facilities, because they usually outsource their testing to different companies. Including details about the testing methods and their reliability on the websites could prove to be beneficial.

Although current clinical practice guidelines do not routinely recommend *APOE* testing in medical practice, it is conceivable that *APOE* testing might become more widely used in the near future. Its use would not only be limited to stratifying patients according to the risk of developing ARIA before starting DMT treatment (Cummings et al. 2023), but could also promote health-related behavioral changes in individuals with the ε4 allele[s] (Marshe et al. 2019), such as managing potentially modifiable risks of AD (e.g., smoking, social isolation) (Livingston et al. 2020), or facilitating the recruitment of at-risk individuals to AD prevention trials (Langbaum et al. 2019; Fockler et al. 2021; Ryan et al. 2021).

Integrating *APOE* testing by DTC services into medical care services may potentially be advantageous, serving as a complement of out-of-insurance *APOE* testing at medical facilities before starting treatment with DMT. In Japan, a single medical facility is prohibited from offering a combination of treatments that are both covered and not covered by public health insurance for the same disease. For such cases, it is essential to provide DTC users with adequate information about *APOE* testing, facilitate their access to genetic counseling upon request, and ensure the quality of sample collection and testing procedures. These are critical concerns that need specific attention and evaluation in the future. This is not only true for the *APOE* testing, but also for other genetic tests available in DTC services.

In conclusion, our analysis revealed that the features of website advertisements varied among individual facilities. Additionally, only about one-third of these facilities had specialists with the appropriate expertise involved in their practices. These results suggest that the self-determination of patients regarding *APOE* testing could be improperly influenced by some medical facilities in Japan, particularly at the initial decision-making stage. Therefore, it is necessary to further discuss how and to what extent information about *APOE* testing should be conveyed to patients through medical facility website advertisements.

### Supplementary Information

Below is the link to the electronic supplementary material.

Supplementary file1 (DOCX 31 KB)

## Data Availability

We will not publicize the data including the list of medical facilities we analyzed to avoid identifiability of the facilities.

## Abbreviations

AD: Alzheimer’s disease
ARIAs: Amyloid-related imaging abnormalities
DMT: Disease-modifying therapy
DTC: Direct-to-consumer
BIC: Bayesian information criterion
LCA: Latent class analysis
