# Supplementary material for: Advertisement by medical facilities as an opportunity route of APOE genetic testing in Japan: a website analysis
Source: J Community Genet. 2024 Jan 16;15(2):195–204. doi: 10.1007/s12687-024-00697-9 (PMC11031550; doi:10.1007/s12687-024-00697-9)
Supplement: Supplementary file 1 — Supplementary file1 (DOCX 31 KB) [file 12687_2024_697_MOESM1_ESM.docx]

**Supplemental Table 1. Result summary of website features, by the type of medical facility**

| Tier - No. | | Feature | Satisfying websites,  in large hospitals (%) | Satisfying websites,  in small clinics (%) | P-value |
| --- | --- | --- | --- | --- | --- |
| 1–1 | Contact information | | 33 (100%) | 187 (100%) | N/A^†^ |
| 1–2 | Details *or* Costs of testing | | 33 (100%) | 175 (93.6%) | 0.221 |
|  | 2–1 | Explaining the *APOE* as a risk gene | 30 (90.9%) | 145 (77.5%) | 0.101 |
|  | 2–2 | Cost clarified | 27 (81.8%) | 127 (67.9%) | 0.149 |
| 1–3 | Major risks *or* adverse reactions | | 22 (66.7%) | 88 (47.1%) | 0.058 |
|  | 2–3 | Notes on genetic testing | 0 (0%) | 1 (0.5%) | 1.000 |
|  | 2–4 | Examination method | 22 (66.7%) | 88 (47.1%) | 0.058 |
| 2–5 | | Notes on interpreting results | 9 (27.3%) | 35 (18.7%) | 0.248 |
| 2–6 | | Appropriate specialists | 25 (75.8%) | 53 (28.3%) | <0.001* |
| 2–7 | | Not external links | 31 (93.9%) | 168 (89.8%) | 0.748 |
| 2–8 | | Referring to genetic counseling | 0 (0%) | 2 (1.1%) | 1.000 |
| 2–9 | | Not covered by health insurance | 31 (93.9%) | 131 (70.1%) | 0.003* |

Abbreviations: N/A, not available.

P-values are results of the Fisher's exact test, derived from a 2-by-2 contingency table that evaluates each feature in relation to the type of medical facilities (i.e., small versus large). For Tier 1–1, all websites met this criterion, rendering the p-value non-applicable (N/A), which is indicated by a dagger [†]. An asterisk (*) denotes a significance level with a p-value of less than 0.05.
